# Supplementary material for: The protective mutation A673T in amyloid precursor protein gene decreases Aβ peptides production for 14 forms of Familial Alzheimer’s Disease in SH-SY5Y cells
Source: PLoS One. 2020 Dec 28;15(12):e0237122. doi: 10.1371/journal.pone.0237122 (PMC7769289; doi:10.1371/journal.pone.0237122)
Supplement: Attachment — Submitted filename: Response to Reviewers.docx [file pone.0237122.s003.docx]

# **Response to reviewers**

**Journal Requirements:**

**Comments to the Author**

**1. If the authors have adequately addressed your comments raised in a previous round of review and you feel that this manuscript is now acceptable for publication, you may indicate that here to bypass the “Comments to the Author” section, enter your conflict of interest statement in the “Confidential to Editor” section, and submit your "Accept" recommendation.**

**Reviewer #1: All comments have been addressed**

**Reviewer #2: All comments have been addressed**

**6. Review Comments to the Author

Please use the space provided to explain your answers to the questions above. You may also include additional comments for the author, including concerns about dual publication, research ethics, or publication ethics. (Please upload your review as an attachment if it exceeds 20,000 characters)**

**Reviewer #1: The Authors have adequately responded to all my comments.**

**Reviewer #2: The manuscript has improved. However, there are still some minor issues that need to be addressed.
Overall, the description of the mutations remains imprecise. Terms like ‘some’, ‘most of’ etc..have to be replaced.
Moreover, it is not clear whether 29 or 30 FAD mutations have been tested:
Figure 3 A-C : overall 30 mutations tested
Figure 4: 29 mutations tested
Figure 5: 30 mutations tested**

An FAD mutation is a mutation changing the normal processing of beta amyloid.

In our study, there are 30 mutations tested. The 30^th^, A673V could not obviously be tested in Figure 4 it is located exactly at the location of A673T. But we wanted to make A673V appear in a matter of comparison in Figure 3 and 5. S you can see in Figure 5, A673V “A673T treated” ration does not appear because it is impossible to get it.

**Minor comments
Title
production for some forms of Familial Alzheimer’s Disease in SH-SY5Y cells
Please replace ‘some’ with a more accurate term**

We made all the corrections. **Abstract
Line 17. The deposition of plaques in the brain leads to the onset and development of Alzheimer’s disease.
Please change to: the deposition of Aβ plaques in the brain
Line 18. The Amyloid precursor protein (APP) is usually cut by alpha-secretase
I would write: the Amyloid precursor protein (APP) is cleaved by alpha-secretase (non-amyloidogenic processing of APP)
Line 22-23. containing APP genes with 29 FAD mutations
Please change it to ‘APP gene’**

We made all the corrections.

**Introduction
Line 72. one APP mutation decreases
Please refer to the specific mutation: one APP mutation (p.A673T)
Line 89. ‘passed away’, please change to ‘deceased’
Line 94. mutation and various FAD mutations: please change to 29 FAD mutations**

We made all the corrections. **Results
Line 106. wild-type APP plasmid (Fig 2). Please specify.
Line 132-134. The reduction of Aβ40 and Aβ42 peptide production by the insertion of the additional A673T Islandic mutation is clear not only for the wild-type APP control gene but also for several FAD mutations.
Please reformulate it**

We reformulated it everything. We hope it is clearer. **Line 120-123. Nearly all of the FAD mutations increased the Aβ40 and Aβ42 concentrations.
The results obtained were consistent with the literature with some exceptions such as the
H677R (English) and D678N (Tottori) mutation, which were reported to only enhance
aggregation and not Aβ peptide accumulation
'Nearly all', please state exactly how many.

Line 126. Fig 3. Plasmids coding for various FAD mutations, please state exactly how many**

**We corrected it.

Line 133-134. The reduction of Aβ40 and Aβ42 peptide production by the insertion of the additional A673T Islandic mutation is clear not only for the wild-type APP control gene but also for several FAD mutations.

Please reformulate it: clear? Significant? several FAD mutations, how many (%)?

Line 138-139. However, the addition of the A673T mutation increased the Aβ40 concentrations for 3 FAD plasmids (10%) and the Aβ42 concentrations for 4 FAD plasmids (14%) FAD mutations

Please list the APP mutations for these 3 and 4 FAD Plasmids

Line 167-168.** **The most encouraging mutation among all the FAD mutations of the APP

Please change the title and write a paragraph only about ‘The London mutation (APP p. V717I)’

Line 168. all the FAD mutations please change it to all the FAD mutations tested**

**We addressed all the comments.

Discussion

Line 183-184. The A673T mutation has been theorized to provide protective effects against AD onset and development [18].

Please, change to ‘The A673T mutation has been shown’

Line 190-191. 14 of the 29 FAD .. 10 of the 29 FAD mutations investigated

Please report (%)

Line 199-200. Another interesting observation of this study was the drastic difference in the presence of Aβ peptides, especially when different mutations of the same codon.

This sentence is not clear, please reformulate it.

Line 202-203. However, the presence of A673T actually increased the formation of both peptides in presence of the I716T mutation.

Please comment on it.

Line 212. All said, for the purposes,

Please reformulate it

Line 219. to strongly reduce of their Aβ peptide levels

Please rephrase it.

Line 220-225. Enrolling pre-symptomatic patients lacking FAD mutations
for clinical trials targeting sporadic Alzheimer patients would likely prove difficult. The large
sample size required for statistical significance in the face of patient reluctance when weighing the risk against the odds of developing Alzheimer’s disease in the first place [28]. That said, the data observed and discussed in this article stands to help validate the launch of clinical trials for carriers of the London mutation (approximately 30 families). As

Please, reformulate it


Line 255-256. Gyorgy et al. have managed to disrupt the APP KM670/671NL Swedish mutation allele using CRISPR [34]

Please rephrase it.

Line 261-263. This study has demonstrated that the insertion of the A673T mutation decreases Aβ40 and Aβ42 production in SH-SY5Y cells and could lead to potential benefits for some forms of Familial Alzheimer’s Disease

To reformulate: A673T mutation decreases Aβ40 and Aβ42 production in SH-SY5Y cells in (how many?) FAD mutations and may lead to…

Line 264-267. in APP affects other genes in trans. More specifically, genes that have been related to AD such as the PSEN1 or PSEN2 genes. It would additionally be worth exploring if A673T could compensate for a weak clearing system that originates from the APOE4 risk factor and therefore allow A673T to treat sporadic AD cases [35].

Please reformulate it.**

**We addressed all the Discussion comments.

Table 1
I would write the ‘Wild type’ in the first raw and try to order the mutations based on the level of significance.**

**We put “wild type” at the top of the table. However, we preferred to let the ordering the original way in order to keep clarity.**
